# Association of vitiligo with immune-checkpoint inhibitor therapy: A systematic review

**DOI:** 10.1016/j.jdin.2024.10.005

**Published:** 2024-11-14

**Authors:** Lauren Fleshner, Katie Roster, Lillian Xie, Shari R. Lipner

**Affiliations:** a School of Medicine, New York Medical College, Valhalla, New York; b Department of Dermatology, Georgetown University School of Medicine, Medstar Washington Hospital Center, Washington, District of Columbia; c Department of Dermatology, Weill Cornell Medicine, New York, New York

**Keywords:** immune-related adverse events, immunotherapy, immunotoxicity, nivolumab, pembrolizumab, vitiligo

*To the Editor:* Immune checkpoint inhibitors (ICIs) have revolutionized cancer treatment. Patients taking ICIs may develop cutaneous immune-related adverse events (cirAEs), with vitiligo being one of the most common in melanoma, but rarely described in other cancers.^1^^,^^2^ Therefore, we performed a systematic review describing ICI-induced vitiligo among oncology patients.

Using Preferred Reporting Items for Systematic Reviews and Meta-analyses guidelines, PubMed, EMBASE, Web of Science, and Cochrane databases were searched for studies describing adult (18+) oncology patients taking ICIs (anti-PD(L)1, anti-CTLA4, or combination) who subsequently developed vitiligo (search strategy available at https://doi.org/10.17632/pd4k6rfr8y.1). Reviews, books, editorials, non-English studies, abstracts, and studies lacking patient-level data were excluded. Articles were independently screened by 2 authors (L.F., K.R.), with discrepancies resolved by discussion until consensus (Fig 1).

**Fig 1:**
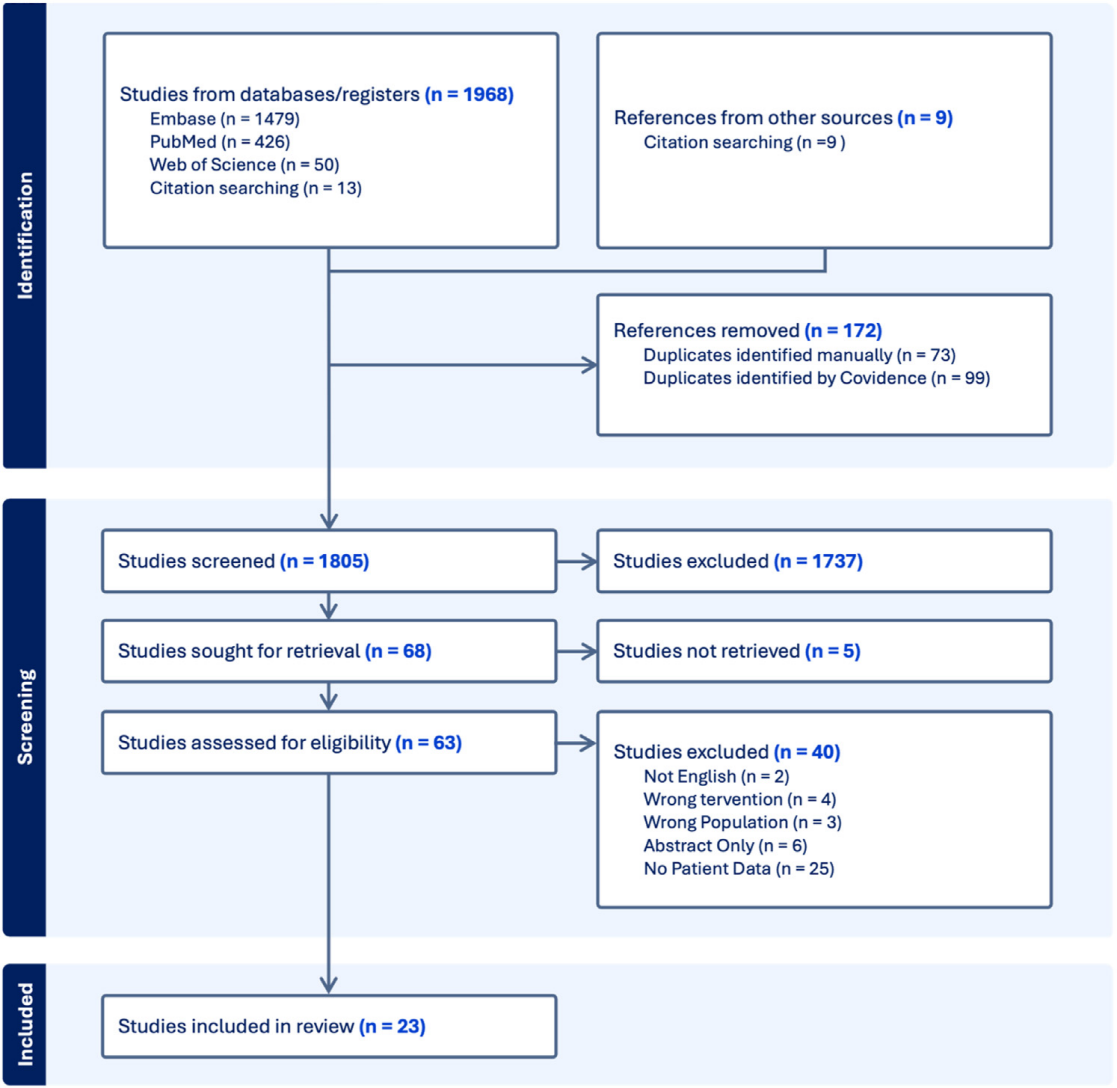
PRISMA flow diagram of included studies.

Generated by Covidence.

Twenty-three articles (*n* = 24 patients) were included for analysis (Table I). Median age of patients was 61.3 years, with a majority of males (62.5%). The most common malignancies were metastatic melanoma (37.5%) and nonsmall cell lung cancer (16.7%). Among ICIs, monotherapy with anti-PD1 inhibitors nivolumab and pembrolizumab were most frequently reported (50.0% and 29.2%, respectively). Median time to vitiligo onset was 12 weeks (range = 1-80) after a median of 3 (range = 1-30) ICI doses. The most common presentation was diffuse vitiligo (66.7%), followed by head/neck (8.3%), and trunk (8.3%). Overall, 66.6% and 53.3% of reported melanoma and non-melanoma patients had complete or partial responses to immunotherapy, respectively.

**Table I:** Reported patient characteristics among included studies

Patient characteristics (*n* = 24)	*N* (%)
Age (median, range)	61.33 (30-79)
Sex	
Male	15 (62.5)
Female	9 (37.5)
Race	
Caucasian	5 (20.8)
Black/African American	2 (8.3)
Asian	1 (4.2)
Indian	1 (4.2)
Not reported	15 (62.5)
Primary cancer site	
Melanoma	9 (37.5)
Nonsmall cell lung cancer	4 (16.7)
Squamous cell carcinoma	3 (12.5)
Renal cell carcinoma	2 (8.3)
Pulmonary adenocarcinoma	2 (8.3)
Soft tissue sarcoma	1 (4.2)
Gastric adenocarcinoma	1 (4.2)
Hepatocellular carcinoma	1 (4.2)
Papillary adenocarcinoma	1 (4.2)
ICI	
Nivolumab (Anti-PD-1)	12 (50.0)
Pembrolizumab (Anti-PD-1)	7 (29.2)
Ipilumumab (Anti-CTLA4)	2 (8.3)
Camrelizumab (Anti-PD-1)	1 (4.2)
Tislelizumab (Anti-PD-1)	1 (4.2)
Combination (Nivolumab/Ipilimumab)	1 (4.2)
Location of vitiligo	
Diffuse/multiple locations	16 (66.7)
Head/neck	2 (8.3)
Trunk	2 (8.3)
Not reported	4 (16.7)
Number of doses to vitiligo (median)	3 (1-30)
Time to onset of vitiligo (median, wk)	12 (1-80)
Response to ICI	
Total cohort (*n* = 24)	
Complete resolution	5 (20.8)
Partial resolution	9 (37.5)
Disease progression	5 (20.8)
Not reported	5 (20.8)
Melanoma (*n* = 9)	
Complete resolution	3 (33.3)
Partial resolution	3 (33.3)
Disease progression	3 (33.3)
Nonmelanoma (*n* = 15)	
Complete resolution	2 (13.3)
Partial resolution	6 (40.0)
Disease progression	2 (13.3)
Not reported	5 (33.3)

*ICI*, Immune chekpoint inhibitor.

We found that a majority of oncology patients treated with ICIs who developed vitiligo as a cirAE had partial or complete response to immunotherapy, which supports the hypothesis of vitiligo portending a favorable outcome.^1^, ^2^, ^3^ In a study of 3731 ICI recipients, 7.1% of melanoma patients and 1.1% of non-melanoma patients developed vitiligo, with vitiligo vs nonvitiligo patients having improved survival (HR: 0.29, 95% CI: 0.12-0.71, *P* = .007).^4^ However, occurrence and prognostic value of vitiligo in nonmelanoma cancers are less established. Our review highlights that vitiligo as a cirAE is also reported in nonsmall cell lung cancer and squamous cell carcinoma ICI-treated patients, with an 80% partial or complete response to immunotherapy.

Median time to vitiligo onset was 12 weeks, suggesting that while it may serve as an early indicator for treatment efficacy, the timelines in which patients present with vitiligo may vary significantly.

Limitations include small sample size, selection bias, lack of long-term outcomes, and lack of higher-quality studies, including randomized trials.

In sum, our findings suggest that vitiligo is a common cirAE typically presenting in patients with melanoma and nonsmall cell lung cancer. In this limited cohort, most patients with ICI-induced vitiligo appeared to show a positive response to ICIs. Identifying patient groups likely to develop vitiligo may help dermatologists stratify their patients on ICIs based on risk profile and possible response to treatment, and tailor follow-up and supportive care accordingly.

Further research is needed to validate these findings among larger cohorts and explore underlying immunologic mechanisms of ICI-induced vitiligo, which could ultimately improve management and patient outcomes in oncology. This information could provide valuable insights into the patient burden of vitiligo and corroborate the association of vitiligo as a prognostic marker.

## Conflicts of interest

Dr Lipner has served as a consultant for Ortho-Dermatologics, Moberg Pharmaceuticals, Eli Lilly, and BelleTorus Corporation. Authors Fleshner, Xie, and Dr Roster have no conflicts of interest to declare.
